# Computational Modelling Approaches on Epigenetic Factors in Neurodegenerative and Autoimmune Diseases and Their Mechanistic Analysis

**DOI:** 10.1155/2015/737168

**Published:** 2015-11-09

**Authors:** Afroza Khanam Irin, Alpha Tom Kodamullil, Michaela Gündel, Martin Hofmann-Apitius

**Affiliations:** ^1^Department of Bioinformatics, Fraunhofer Institute for Algorithms and Scientific Computing, 53754 Sankt Augustin, Germany; ^2^Bonn-Aachen International Center for IT, Rheinische Friedrich-Wilhelms-Universität Bonn, Dahlmannstrasse 2, 53113 Bonn, Germany

## Abstract

Neurodegenerative as well as autoimmune diseases have unclear aetiologies, but an increasing number of evidences report for a combination of genetic and epigenetic alterations that predispose for the development of disease. This review examines the major milestones in epigenetics research in the context of diseases and various computational approaches developed in the last decades to unravel new epigenetic modifications. However, there are limited studies that systematically link genetic and epigenetic alterations of DNA to the aetiology of diseases. In this work, we demonstrate how disease-related epigenetic knowledge can be systematically captured and integrated with heterogeneous information into a functional context using Biological Expression Language (BEL). This novel methodology, based on BEL, enables us to integrate epigenetic modifications such as DNA methylation or acetylation of histones into a specific disease network. As an example, we depict the integration of epigenetic and genetic factors in a functional context specific to Parkinson's disease (PD) and Multiple Sclerosis (MS).

## 1. Introduction

In the 19th century, Gregor Mendel defined the mechanism of inheritance patterns, which laid the ground for genetics in modern biology. However, Mendel's theories could explain neither how different individuals in a population are genetically similar but exhibit different phenotypes, nor how identical twins are prone to different diseases. Recent studies confirmed that copy number variations, single nucleotide polymorphism, or any heritable changes in the DNA sequence could be a plausible additional explanation for Mendel's observation. In 1942, Waddington used the term* epigenotype* as a name for the study of causal mechanisms through which genes exhibit phenotypic effects and their adaptive interaction with the environment [1]. These epigenetic causal mechanisms involve histone modifications, DNA methylation, and abnormal RNA regulation, which can alter normal biological processes by heritable silencing of genes, although they do not cause any nucleotide sequence changes in chromosomal components [2]. Gill published the first paper describing epigenetic mechanism in drosophila egg promorphology [3]. In 1971, Tsanev and Sendov proposed the role of epigenetics in neoplastic transformation and the process of carcinogenesis [4]. Holliday reviewed the methylation of cytosine in DNA and how they are consistent to the levels of gene expression in higher organisms like human, mouse, and hamster [5]. He also illustrated that epigenetic effects are closely linked to aging such that decrease in methylation correlates with lifespan. It has later been demonstrated that epigenetic modifications are tissue-specific phenomena that can have dramatic effects on the silencing, the increase, or the reduction of the expression of genes in a given tissue. Song et al. observed variations of the methylation status in different developmental stages [6]. Additionally, Chen and Zhang showed the risk of neonatal mortality due to maternal vascular underperfusion, which is a result of epigenetic modifications in several genes during pregnancy [7].

Several studies illustrate how nutrition and environmental factors influence epigenetic modifications. A study based on an African-American cohort demonstrated that epigenetic factors like psychological stress and social context are related to inflammation in coronary heart disease and stroke [8]. In the progression of type-2 diabetes mellitus (T2DM), Prattichizzo et al. [9] reviewed interactions between epigenetic (DNA methylation, posttranslational histone modifications, and miRNA regulation) and environmental factors (lifestyle and mainly dietary habits). Duru et al. proposed several dietary chemoprevention agents—such as Retinoids/Vitamin A, Resveratrol, EGCG/Green Tea, and Vitamin D—which act on miRNA-signalling pathways to be novel therapeutics in breast cancer [10].

It is noteworthy that environmental exposures during early stage of life can also induce persistent alterations in the epigenome, which may lead to an increased risk of disease later in life. Reviews by Van Dijk et al. and Cordero et al. investigated different epigenomics patterns in obesity during early and later stage of life [11, 12]. They elucidated the role of dietary supplements and environmental conditions on epigenetic mechanisms during the pregnancy period, which lead to the risk of obesity in offspring.

## 2. Epigenetics in Neurodegenerative and Autoimmune Disease

With the rising momentum of biomedical science, several studies on neurodegenerative diseases (NDDs) not only showed environmental influences on molecular and cellular changes [13, 14] but also established possible relationships between genes and the environment [15]. The major mechanisms for epigenetic alterations found in these diseases include DNA methylation, histone tail modifications, chromatin remodelling, and mechanisms regulated by small RNA molecules [16–18]. Epigenetics in neurodegenerative and autoimmune diseases are of current interest to many researchers and more recently several studies have shed light on the role of epigenetic alterations in autoimmune diseases and NDDs.

Ravaglia et al. discussed the association of folate and Vitamin B12 levels in nutritional diet with the prevalence of NDD [19]. An experiment performed on aged monkeys showed epigenetic changes in APP expression and amyloid beta level due to lead (Pb) exposure [20]. Another study by Baccarelli and Bollati explained how air-pollutants (black carbon, benzene) and toxic chemicals (arsenic, nickel, and diethylstilbestrol) alter gene expression accompanied by epigenetics changes [21]. This paper reviewed all possible metals and chemicals; those are responsible for up- or downregulation of disease specific gene such as BDNF.

Since NDDs are prevalent in the aged population, experiments conducted on NDD patients have revealed how environmental factors such as age, lifestyle, diet, and level of education influence the development of diseases and also highlighted the crosstalk of environmental factors with genes [22]. HDAC gene expression has been shown to be downregulated by Kaliman et al. due to moderate physical activities, which in turn reduce the expression of proinflammatory genes in NDDs [23]. Other than physical exercise, Nicolia et al. reviewed the role of environmental factors such as stressors (physical and behavioral), pesticides, and mental exercise causing DNA methylation in age-related diseases, specifically in AD [24]. The authors suggested that longer lifespan increases the risk of environment-induced epigenetic changes. In a detailed study [25] of epigenetics in AD, decreased DNA methylation was observed in the temporal neocortex of monozygotic AD twins. Manipulation of histone tail acetylation with HDAC inhibitors also has been investigated in several animal models of AD [26]. Martí et al. have explained a set of deregulated miRNAs that participate in altered gene expression in neurodegeneration, especially in Huntington's disease [27].

A hypothesis, namely, “hapten hypothesis,” was introduced by Mintzer et al. in 2009, which describes that drugs like Penicillin and Clozapine play the role as haptens to produce antibodies against neutrophils in case of autoimmune diseases, such as Systemic Lupus Erythematosus (SLE) [28]. Uhlig et al. mentioned smoking as risk factor in addition to age and gender in another systemic autoimmune disorder, that is, Rheumatoid Arthritis (RA) [29]. Similarly, ultraviolet radiation also alters the immune mechanisms that may result in Lupus Erythematosus (LE) [30]. From the above discussion it is evident that epigenetic factors play a significant role in the context of NDD and autoimmune disease.

**Figure 1:**
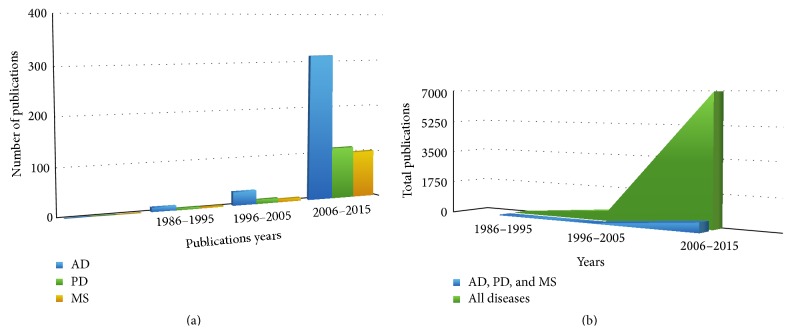
(a) Statistics over scientific publications around epigenetics related neurodegenerative (AD and PD), autoimmune diseases and other diseases using PubMed with queries ((“Parkinson's disease”) AND epigenetics), ((“Alzheimer's disease”) AND epigenetics), and ((“Multiple Sclerosis”) AND epigenetics), last accessed on 7/20/2015. In (a), blue, green, and orange coloured bars represent the total number of publications, for AD, PD, and MS, respectively. (b) This figure illustrates the trend of research on other diseases around epigenetics compared to NDD (AD and PD) and autoimmune (MS) disease, where green coloured portion representing the studies on all sorts of diseases and blue portion covers only AD, PD, and MS related researches.

Although there is growing interest in epigenetics of NDDs and autoimmune diseases, only a few studies have been performed specifically on PD and MS. In fact, only a very limited number of studies deal with the functional consequences of epigenetic modifications and perturbed mechanisms leading to a particular phenotype. A systematic comparison of the number of epigenetic studies in AD, PD, and MS in the last years is shown in Figure 1(a). The graph shows that the number of scientific publications on epigenetics in PD and MS is significantly lower than the number of papers on epigenetics in AD.  Figure 1(b) represents the overall trend in epigenetic studies; it becomes obvious that AD, PD, and MS represent only a minority fraction of the literature on epigenetics mechanisms, in particular when compared with the predominant indication areas arthritis, cancer, and diabetes.

## 3. Computational Modelling of Epigenetic Factors in a Functional Context

To represent, manipulate, and visualize large amounts of biological data from different sources, computational modelling has become an intuitive approach. Artyomov et al. proposed an “epigenetic and genetic regulatory network” that describes how transcription factors affect cellular differentiation by reprogramming embryonic cells [31]. Irrespective of any specific disease context, a computational micromodel for epigenetic mechanisms was developed by Raghavan et al., demonstrating the interaction of histone modifications with DNA methylation and transcription process [32]. The model was able to identify the transcription rate when the level of DNA methylation is known.

From high throughput gene expression data of 12 human cell lines, a model integrating transcriptomic data and histone modification has been developed, called Epigenetic Regulatory Network [33], which identifies the main contributing epigenetic factors among different cell types. To facilitate the systematic integration of High Throughput Sequencing (HTS) epigenetic data, Althammer et al. have described a new computational framework. This workflow was inspired by machine learning algorithms and can be used to find alterations of epigenetic states between two given cell types [34]. Artificial Epigenetic Regulatory Network (AERN) proposed by Turner et al. has included DNA methylation and chromatin modification as the epigenetic elements in addition to genetic factors. They showed an example of how disease specific genes can be allocated in the network according to environmental changes and how gene expression regulation can be analysed within the network [35]. In a recent review paper [36], Hidden Markov Models (HMM) have been used to handle the complexity of epigenetic mechanisms, especially different patterns of DNA methylation. For autoimmune diseases, Farh et al. developed an algorithm, named “Probabilistic Identification of Causal SNPs (PICS),” which was able to find out the possibility of SNPs to be causal variants in immune cell enhancers when epigenetic modifications on that chromatin site are known [37].

Although there are algorithms that identify epigenetic modifications, there are no previous evidences describing the interpretation of functional consequences of epigenetic modifications in disease mechanisms. Here, we propose a computer-readable modelling strategy that is competent of fusing knowledge and data based information, which is capable of explaining the functional consequences of epigenetic modification in a mechanistic fashion. In this paper, we introduce the Biological Expression Language (BEL; http://www.openbel.org/) that is the main base of building models for epigenetics analysis of PD and MS.

BEL integrates literature-derived “cause and effect” relationships into network models, which can be subjected to causal analysis and used for mechanism-based hypothesis generation [38]. The semantic triple-based modelling language used here enables the application of Reverse Causal Reasoning (RCR) algorithms, which support the identification of mechanistic hypotheses from the corresponding causal network. The RCR methodology allows for investigating to what extent a knowledge-based set of triples is supported by omics data (e.g., gene expression data); the method is therefore suited for inference based on qualitatively significant data [39]. To enable a quantitative assessment and to perform comparative mechanistic analysis, another algorithm is integrated in the BEL framework: the Network Perturbation Amplitude (NPA) method. Although it uses the same network structure like RCR, its main purpose is to estimate the activity changes of a specific biological process when a pathophysiology state is compared to a nonperturbed condition [40].

Until now, BEL based network modelling approaches have been used in various applications such as early patient stratification, biomarker identification [41], and personalized drug discovery [42] in the context of cancer research by different groups. Our objective behind this computational modelling approach aims at harvesting relevant scientific knowledge from unstructured text and to systematically understand the functional impact of epigenetic modification in the context of PD and MS using BEL.

## 4. Role of Epigenetics in Parkinson's Disease Using BEL Models

PD is characterized by a loss of midbrain dopaminergic neurons leading to motor abnormalities and autonomic dysfunctions [43]. Genes such as* SNCA*,* parkin*,* PINK1*, and* FBX07* have been identified to be responsible for pathophysiological mechanisms like mitochondrial damage, repair, and oxidative stress [17]. There are evidences suggesting that the above-mentioned key genes are epigenetically modified under disease conditions. For example, studies in familial as well as sporadic PD patients suggested that demethylation of the* SNCA* gene stimulates its upregulation [17, 44, 45]. Increasing amounts of* CYP2E1* have been found to promote the formation of toxic metabolites, which further degenerate the dopaminergic neurons [46]. Abnormal epigenetic modifications involved in the pathogenesis of PD have been studied by Feng et al.; in that study, detailed insights on DNA methylation and histone acetylation mechanisms and their association with the disease are reported [47].

To construct an epigenetics model for PD, we have made use of SCAIView (http://bishop.scai.fraunhofer.de/scaiview/), a literature mining environment to extract all relevant articles using the query ([*MeSH Disease: “Parkinson Disease”*])* AND* ([*Parkinson Ontology*: “*Epigenetics*”]). Based on this literature mining approach, we have manually selected 78 articles, which were found to contain relevant information about PD epigenetics. The content of these publications was subsequently encoded in BEL. The model consists of 235 nodes and 407 edges representing 339 BEL statements. The nodes contain 67 proteins/genes, 21 biological processes, 6 SNPs, 3 complexes, 24 chemical entities, 26 miRNAs, and 88 other nodes representing translocation, degradation, and association functions.

**Figure 2:**
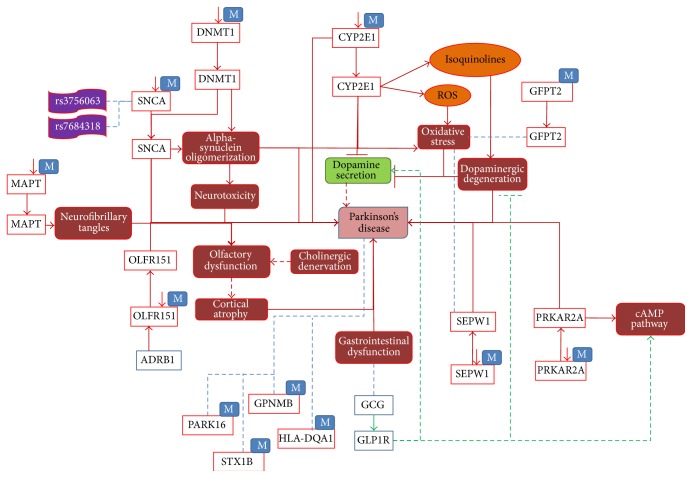
The role of epigenetics modification; hypomethylation around certain genes in PD. In this figure, red lines indicate the disease state interactions and green lines show normal state. Blue lines show the association between entities with unknown direction. Dotted lines are the interpretation, which needs to be further analysed. “M” associated with a gene entity denotes a methylation process and down-arrows besides represent decreased methylation.

As shown in Figure 2, seven representative genes, namely,* SNCA*,* MAPT*,* DNMT1*,* CYP2E1*,* OLFR151*,* PRKAR2A*, and* SEPW1*, were reported to be hypomethylated under disease conditions. In these cases, hypomethylation causes overexpression of genes that perturb normal biological processes. Increased expression of* SNCA* and* DNMT1* caused by decreased methylation of these genes results in alpha-synuclein oligomerization, which in turn causes neurotoxicity in PD [48]. Along with that, two SNPs, rs3756063 and rs7684318, were associated with hypomethylation of SNCA in PD patients. Similarly, the* CYP2E1* gene was detected to be upregulated due to (i) hypomethylation, (ii) release of isoquinolines, and (iii) Reactive Oxygen Species (ROS), which lead to dopaminergic degeneration and oxidative stress, respectively [49]. Increased neurofibrillary tangles in PD have been reported to be linked with high expression of* MAPT* gene, as a consequence of reduced methylation [50]. Furthermore,* ADRB1* induced the hypomethylation of the* OLFR151* gene [51]. As a result, overexpression of* OLFR151* leads to olfactory dysfunction and cortical atrophy, which are early symptoms of PD [52].

GWAS and epigenomic studies suggest that* SEPW1* and* PRKAR2A* were overexpressed due to hypomethylation in PD patients [53]. However, there is lack of well-established knowledge about the functional role of* SEPW1* and* PRKAR2A* in the context of PD. We identified only one study that reports the association of* SEPW1* with PD brains [53]. Similarly, we did not find any direct biological consequences of* PRKAR2A* to play a role in the disease state. We employed a dedicated data mining approach in our model and identified the association of* PRKAR2A* with the cAMP pathway. It has been found that cAMP signal transduction pathway is stimulated by* GCG* (glucagon) [54] and its receptor* GLP1R,* which is secreted by the gastrointestinal mucosa [55].* GLP1R* is also known to play a role in dopamine secretion and inhibiting dopaminergic degeneration [56]. Therefore we speculate that gastrointestinal dysfunction (an early symptom of PD) may result in a perturbation of the cAMP pathway and that this could be a possible mechanistic link to hypomethylation of* PRKAR2A* in PD. In addition to the above-mentioned hypomethylated genes, five more methylated genes were identified in the PD context, namely,* GFPT2*,* GPNMB*,* PARK16*,* STX1B*, and* HLA-DQA1*, where only* GFPT2* was inferred to be associated with oxidative stress [57]. These examples demonstrate that even though the analysis of high throughput data like GWAS or epigenetic studies do predict many disease-associated risk genes, no further research has been carried out to understand the functional impact of these genes.

**Figure 3:**
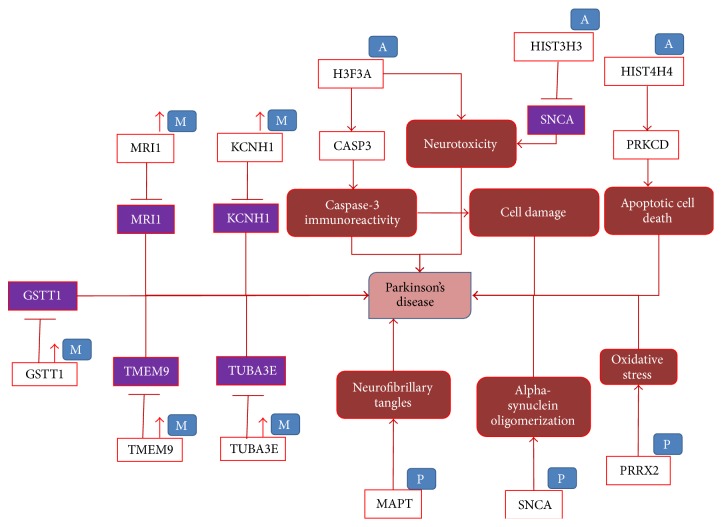
The role of epigenetics modification, hypermethylation, phosphorylation, and acetylation around certain genes in PD. In this figure also red lines indicate the disease state interactions. “M” associated with a gene entity denotes a methylation process and up-arrows besides represent an increase of methylation. “P” and “A” represent the phosphorylation and acetylation processes, respectively. Genes in purple boxes denote lower expression of genes.

In addition to Figure 2, we represent in our modelling approach three more highly relevant epigenetics modifications, namely, hypermethylation, phosphorylation, and acetylation (Figure 3). Five genes,* GSTT1*,* MRI1*,* KCNH1*,* TMEM9*, and* TUBA3E*, were reported to be significantly hypermethylated resulting in low expression of genes [58]. However, there were no studies describing the functional role of these genes in the PD context. In case of acetylation modification,* H3F3A*,* HIST3H3*, and* HIST4H4* were shown to be acetylated under disease conditions. Acetylated* H3F3A* increases* CASP3* activity and thereby may cause cell damage [59]. Acetylation in* HIST3H3* decreases the expression of* SNCA* leading to neurotoxicity [60], whereas* HIST4H4* acetylation induces the activity of* PRKCD*, which promotes apoptotic cell death [59]. Phosphorylation of* MAPT*,* SNCA*, and* PRRX2* causes deposition of neurofibrillary tangles, alpha-synuclein oligomerization, and oxidative stress, respectively, in PD [50, 61].

The enlisted microRNAs in Table 1 were suggested to regulate the epigenetic modification in disease state of Parkinson. These microRNAs bind to their target and downregulate or upregulate their expression in diseased condition. For instance,* MIR34C* induces the expression of the* PARK7* gene, which in turn causes oxidative stress in PD. Some microRNAs function together (i.e.,* MIR34B* and* MIR34C*) while others target individually specific genes such as* PARK7*,* PARK2*, and* TP53* to cause dysregulation in target genes, which may contribute to the disease aetiology [62].

## 5. Role of Epigenetics in Multiple Sclerosis Using BEL Models

Multiple Sclerosis, a complex autoimmune disease of the central nervous system, is characterized by inflammation, demyelination, and destruction of the axons in the central nervous system [63]. Although the aetiology is not known, there is accumulating evidence that, in a cohort with genetic predisposition, environmental factors may play a key role in the development of the disease [64]. Epigenetic studies of this autoimmune disease have shown that disorders of epigenetic processes may influence chromosomal stability and gene expression, resulting in complicated syndromes [65, 66]. In a more detailed study, increased immunoreactivity for acetylated histone H3 in oligodendrocytes was found in a subset of MS samples [67]. Various microRNAs have been shown to differentially express in MS samples; particularly* MIR223* was found to be upregulated in MS patients compared to healthy controls [68]. Major epigenetic mechanisms involved in MS have been listed in a current review article [69], for example, DNA methylation, histone citrullination, and histone acetylation.

Similar to the approach taken with the PD model, we have started with a systematic literature analysis using SCAIView. We extracted information from all articles that could be retrieved with the query ([*MeSH Disease*: “*Multiple Sclerosis*”])* AND* ([*Multiple Sclerosis Ontology*: “*Epigenetics*”]). An overall number of 75 highly relevant articles were used to build the BEL model for MS epigenetics. From this corpus of relevant literature, we have extracted 339 BEL statements to develop a network comprising 215 nodes and 536 edges. The nodes consist of 69 proteins/genes, 43 biological processes, 8 complexes, 18 chemical entities, 38 miRNAs, 8 protein families, and 31 other entities representing translocation, degradation, and association functions.

**Figure 4:**
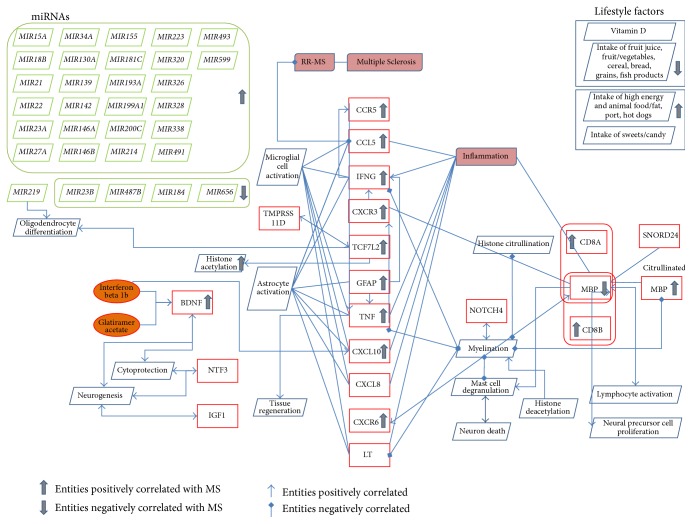
Model around epigenetic factors in Multiple Sclerosis. The figure shows the different epigenetic factors which regulate MS pathology including miRNAs represented in green colour boxes and genes in red colour boxes, chemicals in orange colour, and bioprocess in blue rhombus shape.

Most frequent epigenetic factors affecting MS were found to be miRNA regulation, histone citrullination, and lifestyle factors. We found 24 miRNAs that positively regulate the pathogenesis of MS and* miR23B*,* miR487B*,* miR184,* and* miR656* seem to be less expressed in the diseased context [70]. Apart from these, many epigenetics modifications like acetylation and citrullination were found in cytokines (*IFNG*,* TNF*) [71], chemokines (*CCR5*,* CCL5*,* CXCR3*,* CXCL10*,* CXCL8*, and* CXCR6*) [72], neurotrophic factors (*BDNF*,* NTF3*) [73], surface antigens (*CD8A*,* CD8B*) [74], and other genes like* GFAP*,* MBP*,* SNORD24*, and* NOTCH4.* In addition, dietary factors such as Vitamin D, intake of fruit juice, fruit/vegetables, cereal, bread, grains, and fish products reduce the risk of MS whereas intake of high energy and animal food such as fat, pork, hot dogs, and sweets increase risk of the disease (Figure 4).

## 6. Discussion

Epigenetics is a major mechanism that accommodates gene-expression changes in response to gene-environment interactions. In the last few decades, it has been shown that epigenetic factors play an important role in neurodegenerative as well as in autoimmune diseases. Even though there are strategies to identify new epigenetic modifications, there are very few studies, which link these alterations in DNA to the aetiology of the disease. Given the complexity and the wide variety of entities like epigenetic modifications and genetic variants, which perturb normal biological processes, we need new strategies to integrate data driven and knowledge driven approaches to unravel the mechanisms behind these complex diseases. We demonstrated that it is possible to collectively capture disease-related, epigenetic knowledge and integrate it into a functional context using the modelling language BEL. An adaptation of the BEL syntax enables us to integrate epigenetic modification information like methylation (hypo and hyper), acetylation, phosphorylation, and miRNAs regulation into a specific disease network. In addition to these mechanisms, we have also included the role of many environmental factors such as food habit and obesity to the model which are responsible for the epigenetic modifications.

Although fewer studies related to PD and MS around epigenetics have been published until now, we tried to integrate all available knowledge from the scientific literature. In the case of PD, the main genes which are epigenetically regulated through methylation are* SNCA*,* PARK6*,* CYP2E1*,* PINK1*,* BDNF*,* FGF, MAPT*,* MTHFR*,* OLFR 151*,* PARK16*,* PARK2*,* PARK7*,* TPPP*,* PDE4D*, and* METRNL*. Also we have found acetylation in* H3F3A*,* HIST3H3*, and* HIST4H4* genes and phosphorylation in* MAPT*,* SNCA*, and* PRRX2* genes as major epigenetic modifications in PD along with miRNA regulation. Similarly for MS, we have found several citrullinated or acetylated cytokines, chemokines, transcription factors, neurotrophins, and many dietary factors, which can influence disease processes.

Some of the genes identified are well studied, but for others still an in-depth analysis is needed. Since there are no studies published on these novel candidates derived from data driven approaches, we were not able to link the functional impact of epigenetic modifications to the disease aetiology. For instance, there are about 30 GWAS studies associating the* PARK16* gene with PD, but no detailed information about the functional context of* PARK16* in the pathophysiology of PD exists in the literature. We observe a clear bias towards well-known candidate genes like* SNCA* for PD and* MBP* for MS; in order to overcome this bias, dedicated effort towards investigating the role of the new candidate genes and related bioprocesses is required.

Although BEL has the capability to integrate different biological entities and modifications at the levels of proteins, the current version of BEL is not efficient in representing epigenetic modifications at gene level, so that it is not yet possible to reason over epigenetic effects automatically (e.g., using RCR). It is obvious that we need to extend the syntax of the modelling language in order to formally represent this type of variation and develop algorithms that assess the functional impact based on biological network models.

## Figures and Tables

**Table 1:** Role of microRNAs in PD epigenetics. 26 microRNAs have been identified that have been reported to control PD pathways. Positive and negative correlations of these microRNAs with PD mean if they are inducing or inhibiting the disease state, respectively. Also, we have enlisted the target genes for retrieved microRNAs.

Role of microRNAs in PD epigenetics		
MicroRNA	Relation to PD	Target
*MIR133B*	Negative correlation	*PITX3*
*MIR1*	Negative correlation	*TPPP, BDNF*
*MIR29A*	Negative correlation	—
*MIR221*	Negative correlation	—
*MIR222*	Negative correlation	—
*MIR223*	Negative correlation	—
*MIR224*	Negative correlation	—
*MIR30A*	Positive correlation	*SLC6A3, FGF20, GRIN1, GRIA1*
*MIR16-2*	Positive correlation	*FGF20*
*Mir26a-2*	Associated	*Gria1, Tyr*
*MIR886*	Positive correlation	—
*MIR133B*	Negative correlation	—
*MIR433*	Negative correlation	*FGF20*
*MIR7-1*	Negative correlation	—
*MIR7-2*	Negative correlation	—
*MIR-7*	Positive correlation	*SNCA*
*MIR34B*	Positive correlation	*PARK7, PARK2, TP53*
*MIR34C*	Positive correlation	*PARK7, PARK2, TP53*
*MIR219A1*	Negative correlation	*GRIN1, CD164*
*MIR219A2*	Negative correlation	*GRIN1, CD164*
*MIR124-1*	Positive correlation	*PPP1R13L*
*Mir219a-1*	Negative correlation	*Grin1*
*Mir219a-2*	Negative correlation	*Grin1*
*Mir124a-1*	Negative correlation	—
*Mir124a-2*	Negative correlation	—
*Mir124a-3*	Negative correlation	—

## Abbreviations

BEL: Biological Expression Language
PD: Parkinson's disease
MS: Multiple Sclerosis
AD: Alzheimer's disease
NDDs: Neurodegenerative diseases
T2DM: Type-2 diabetes mellitus
DNA: Deoxyribonucleic acid
RNA: Ribonucleic acid
HDAC: Histone deacetylase
APP: Amyloid Precursor Protein
SLE: Systemic Lupus Erythematosus
RA: Rheumatoid Arthritis
HTS: High throughput sequencing
AGRN: Artificial Gene Regulatory Network
AERN: Artificial Epigenetic Regulatory Network
HMM: Hidden Markov Model
SNP: Single nucleotide polymorphism
ROS: Reactive Oxygen Species
GWAS: Genome-Wide Association Study
cAMP: Cyclic adenosine 3′,5′-monophosphate
miRNA: MicroRNA.
